# Acknowledgement to Reviewers of *Antioxidants* in 2019

**DOI:** 10.3390/antiox9010081

**Published:** 2020-01-17

**Authors:** Antioxidants Editorial Office

**Affiliations:** MDPI, St. Alban-Anlage 66, 4052 Basel, Switzerland

The editorial team greatly appreciates the reviewers who have dedicated their considerable time and expertise to the journal’s rigorous editorial process over the past 12 months, regardless of whether the papers are finally published or not. In 2019, a total of 661 papers were published in the journal, with a median time to first decision of 15 days and a median time from submission to publication of 36 days. The editors would like to express their sincere gratitude to the following reviewers for their generous contribution in 2019:
Abenavoli, LudovicoLiapi, CharisAbourashed, EhabLiby, KarenAcosta-Motos, Jose RamónLichtenberg, DovAdams, James DavidLidon, FernandoAdunyah, Samuel E.Lim, Kyung-MinAgarwal, MukeshLim, SoyeonAgarwal, PujaLin, Hai-ShuAgic, DejanLin, Zuan-TaoAgnieszka, SzopaLindberg, GregerAhn, Dong UkLinderborg, KaisaAhotupa, MarkkuLinsenmeier, Robert A.Aiello, FrancescaLiobikas, JuliusAkagi, SatoshiLiou, Ying-mingAkbar, MohammedLiu, Chia-ChiAkita, SadanoriLiu, JunAlappat, BindhuLiu, YongliangAl-Horani, Rami A.Liverani, ElisabettaAllegra, MarioLivrea, Maria AntoniaAlmajano, María PilarLiwinienko, GrzegorzAlvarez Fernandez, Maria AntoniaLizard, GérardAlvarez-Suarez, JoseLjubenkov, IvicaAmarowicz, RyszardLo Scalzo, RobertoAmorati, RiccardoLoboda, AgnieszkaAncìn-Azpilicueta, CarmenLobysheva, IrinaAngelino, DonatoLodyga-Chruscinska, ElzbietaAngelone, TommasoLogan, MargaretAngelova, AngelinaLognay, GeorgesAngius, FabrizioLohan, SilkeAnifandis, GeorgeLoira, IrisAnraku, MakotoLoizzo, Monica RosaAntelmann, HaikeLombardi, GiovanniAntognelli, CinziaLopes, GracilianaAntonia Kaltsatou, AntoniaLopez, VictorAntonio, MiceliLopez-Escamez, Jose A.Apone, FabioLord, TessaArchibong, AnthonyLorenzetti, StefanoArciello, AngelaLorenzo, Jose M.Armeni, TatianaLovatt, MatthewAroca, AngelesLovejoy, DavidArrebola, Francisco J.Low, MitchellArsene, Andreea LetitiaLowe, GordonArteaga, Jesus F.Lu, SizhaoAshry, MohamedLucarini, MassimoAslam, MuhammadLui, Edmund M.K.Asperger, DanijelaLung, Ming-YeouAston, Kenneth I.Lunov, OlegAtalay, MustafaLykkesfeldt, JensAtkinson, JeffreyLynch, Sean M.Augusto, Jean-FrançoisM. Pedrosa, MercedesAvato, PinarosaM. Vorob'ev, MikhailAxiotis, EvangelosMabou Tagne, AlexAzzini, ElenaMaciejczyk, MateuszBachor, RemigiuszMacmillan-Crow, Lee AnnBacskay, IldikoMadaro, LucaBaczek, KatarzynaMadhukar, BurraBae, JiyoungMaher, PamelaBailey, Charles GMaier, PatrickBajerova, PetraMaietti, AnnalisaBakshi, RachitMaio, Roberto DiBaldisserotto, AnnaMaione, FrancescoBalligand, Jean-LucMaity, ShuvadeepBallmann, ChristopherMajewski, MichalBanerjee, AditiMajid, DewanBanerjee, KalpitaMak, IvanBanti, Christina N.Makedou, KaliBao, YongpingMalapelle, UmbertoBaranova, AnchaMandal, Mihir KumarBaratto, Maria CamillaMandalari, GiuseppinaBarbaric, MonikaMandrich, LuigiBarbieri, AntonioMandrioli, RobertoBarcena, Jose AntonioManjunath, ManuboluBardi, GiuseppeMankin, Richard W.Barisic, LidijaMano, Jun’ichiBarrajon, EnriqueMantell, CasimiroBarreca, DavideManville, RianBarreiro, Sonia LosadaMarc, GabrielBarreto, Maria CarmoMarchetti, AlessandraBarritt, GregMarchiani, SaraBarszcz, MarcinMarchini, CristinaBarta, CsengeleMarechal, AlexandreBartelt, AlexanderMaret, WolfgangBartesaghi, RenataMari, MicheleBartosz, GrzegorzMarincas, OlivianBartoszek, AgnieszkaMarranzano, MarinaBarygina, VictoriaMarsboom, GlennBasak, Saroj K.Marsolais, FredericBasar, MuratMartínez-Espinosa, Rosa MaríaBasile, AdrianaMartinez-Pastor, FelipeBasini, GiuseppinaMartín-Gil, JesusBaskaran, RathinasamyMartini, DanielaBata-Csorgo, ZsuzsannaMartins, Alice M.Batinic-Haberle, InesMartins, NataliaBattaglino, RicardoMarverti, GaetanoBattino, MaurizioMasek, AnnaBauer, GeorgMastanjevic, KristinaBaumann, AnjaMastinu, AndreaBazan, EulaliaMasullo, MariorosarioBazwinsky-Wutschke, IvonneMateus, Maria L.Beberok, ArturMathieu, CélineBednar, PetrMatkowski, AdamBednarski, Patrick J.Matosiuk, DariuszBeharry, Kay D.Matsufuji, HiroshiBekhit, Alaa El-Din AMatsumoto, YasuhikoBeltowski, JerzyMatsumoto, YoshinoriBenarba, BachirMattioli, RobertoBendich, AdrianneMattison, Christopher P.Benson, DavidMay, JamesBentel, Jacqueline M.McAllister, MattBernabo, NicolaMcBean, Gethin J.Bernardini, GiuliaMcCarthy, PumtiwittBernini, RobertaMcDonald, DeniseBernocchi, GraziellaMcmorrow, TaraBerry, Ann AndersonMcPherson, Nicole O.Berti, FedericoMeaney, SteveBevilacqua, AntonioMehla, JogenderBevilacqua, ArturoMeier Zeman, DanielBiagini, GiuseppeMeinke, MartinaBienboire Frosini, CecileMekinic, Ivana GeneralicBijak, MichalMellado, José Miguel RodríguezBilban, MartinMendez, LucíaBiondi, AntonioMendez-Álvarez, EstefaniaBiondi, StefaniaMeneguzzo, FrancescoBirsa, Lucian MMenghini, LuigiBlando, FedericaMenichetti, StefanoBlasi, FrancescaMennerich, DanielaBlazevic, IvicaMerah, OthmaneBloksgaard, MariaMerino, Jose JoaquinBoard, MaryMesaros, Clementina A.Bobis, OtiliaMessina, ConcettaBodea, LiviuMessner, BarbaraBoggia, RaffaellaMezes, MiklosBöhm, VolkerMiceli, AlessandroBohn, TorstenMiceli, AntonioBojić, MirzaMichalke, BernhardBoo, Yong ChoolMichalska, AnnaBooth, BrianMichel, MaximilianBorer, Katarina T.Michels, Alexander J.Borges Dos Santos, Rui M.Mieyal, John J.Bosca, LisardoMiguel, Maria da Graça CostaBosch, ChristineMikulic-Petkovsek, MajaBosch, RicardoMikulski, DamianBourdon, EmmanuelMilardi, DaniloBourgonje, ArnoMilcarek, ChristineBoutin, JeanMilella, LuigiBovera, FulviaMilkovic, LidijaBradshaw, PatrickMiller, IngridBrasili, ElisaMin, HyeyoungBrighenti, VirginiaMinato, Ken-ichiroBrncic, MladenMinkiewicz, PiotrBroderick, Tom L.Miranda, CristobalBrooke, GregMitakou, SofiaBrown, DennisMitjans Arnal, MontserratBrown, LindsayMiyamoto, KojiBrunet, ChristopheMiyamoto, ToshinobuBruno, FerdinandoMiyanaga, AkimasaBuchet, ReneMizobata, TomohiroBucolo, ClaudioMladěnka, PremyslBudryn, GrażynaMnif, WissemBueno Dalto, DanyelMobbili, GiovannaBullon, PedroMocan, AndreiBurger, PaulineMocibob, MarkoBurkey, KentModica, MariaBuschbeck, MarcusMohamed, BashirBusinaro, RitaMohanty, Joy G.Buszewski, BoguslawMOK, Chee KengButts, ChrissieMoldzio, RudolfC Beitz, DonaldMolendijk, Marc L.Caballero-Garcia, BeatrizMolinari, SusannaCabot, CatalinaMollace, VincenzoCabrera Fuentes, Hector A. CabreraMollica, AdrianoCaccamo, DanielaMontesano, DomenicoCacciola, FrancescoMora, AnaCaddeo, CarlaMoretti, RitaCadiz-Gurrea, María De La LuzMorimoto, TatsuyaCairo, GaetanoMorita, ToshisukeCairrao, ElisaMorrell, Jane M.Calokerinos, Antony C.Morris, GordonCamara, Amadou K.S.Morris, Peter C.Camins, AntoniMorton, DavidCammisotto, VittoriaMoschopoulou, EkateriniCampbell, Fiona M.Mosqueira, MatiasCampiglia, PietroMot, AugustinCamporesi, EnricoMotohashi, KenCandela, Diaz-CanestroMotterlini, RobertoCanini, AntonellaMrowczynska, LucynaCanonico, BarbaraMuceniece, RutaCanton, MarcellaMuhlenhoff, UlrichCao, XuMulinacci, NadiaCapasso, RaffaeleMuller, Patricia A. J.Caporaso, NicolaMulloy, BarbaraCapozzi, VittorioMuniz, PilarCapperucci, AntonellaMurase, SachikoCaputo, GregoryMurray, MargaretCarballo, IriaMurtaugh, MaureenCardenia, VladimiroMuszynski, SiemowitCardoso, SusanaMutus, BulentCarlisi, DanielaMysliveček, JaromírCarnevale, RobertoNagayama, YujiCaro, YanisNagy, PeterCarradori, SimoneNair, VimalCarrera, Ceferino A.Nakamaru-Ogiso, EikoCaruso, GianlucaNakamura, SoichiroCarvalho, DanielNakazawa, MitsuruCarvalho, Isabel S.Namasivayam, VigneshwaranCasal, SusanaNardi, MonicaCasalino, ElisabettaNaro, FabioCaser, MatteoNatoli, Riccardo CarloCasini, GiovanniNaumovski, NenadCastellano, ImmacolataNawirska-Olszanska, AgnieszkaCastneiras, AlfonsoNaznin, Most TaheraCastro-Vargas, Henry I.Nazzaro, FilomenaCatala, MyriamNeilson, AndrewCatalano, AntoninoNenadis, NiknenCatauro, MichelinaNeunert, GrazynaCelano, LauraNeves, Adriana CunhaCenac, NicolasNewcombe, GeorgeCerda, BegonaNg, RayCervellati, CarloNguyen, Quang D.Chaimbault, PatrickNicolai, MarisaChang, Jer-MingNicoletti, FerdinandoChang, Jia-FengNiero, GiovanniChang, Ling-ChuNieto, GemaChang, Te-ShengNiki, EtsuoChatterjee, JhinukNikolakaki, EleniChatzidimitriou, EffimiaNimse, Satish BalasahebChaudhuri, DipayanNincevic Grassino, AntonelaChecconi, PaolaNitulescu, George MihaiCheemarla, Nagarjuna ReddyNiwano, YoshimiChen, Chun-JungNjus, DavidChen, Jing-HsienNobile, StefanoChen, Liang-YuNociari, MarceloChen, Shuen-EiNomikos, TzortzisCheng, Juei-TangNorambuena, FernandoCheng, Yuan-BinNorberg, ErikChern, Chi-LiangNovak, LajosCheung, BarryNowicka, PaulinaChiaramello, AnneNunez, OscarChiu, Yi-HanNuzzo, DomenicoChlichlia, KaterinaO’Rear, EdgarCho, Jae YoulObana, AkiraCho, Je-YoelOberley, RebeccaChobot, VladimírOdero-Marah, ValerieChohan, MagaliOkazawa, AtsushiChoi, Jae SueOláh, AttilaChoi, JonghoonOlek, RobertChuyen, Hoang V.Omar, Syed HarisChyau, Charng-CherngOmasa, TakeshiCiaccio, MarcelloOndrias, KarolCiarcia, RobertoOng, Eng ShiCiarlo, LauraOniga, IlioaraCicek, Serhat SezaiOniszczuk, AnnaCicero, ArrigoOracz, JoannaCilla, AntonioOrian, LauraCillero-Pastor, BertaOrlando, GiustinoCimmino, GiovanniOroian, Mircea AdrianCioffi, FedericaOstacolo, CarmineCiofi-Baffoni, SimoneOstuni, MarianoCione, ErikaOthman, Eman M.Cipak Gasparovic, AnaOtto, AngelaCiz, MilanOwczarek, AleksandraClanchy, FelixOzaita Mintegui, AndresClarke, NeilPace, PaulCocco, RaffaellaPadhi, EmilyCoe, ShellyPadilla-Benavides, TeresitaColangelo, Anna MariaPadmanabhan, JagannathCole, MarshaPagano, AlessandraColeman, MichaelPagano, MariaColeman, Mitchell C.Page, MelissaColes, LisaPaixao Coelho, Jose AugustoColobatiu, LioraPak, Jhang HoConde, Sílvia VilaresPalla, FrancoConnelly, Philip W.Pallardó, Federico V.Constantinou, ViolettaPaller, Channing JudithContardi, MarcoPallottini, ValentinaConte, LanfrancoPalma, JoséContino, MarialessandraPalma, MiguelContreras, María Del MarPan, Tai-longCooperstone, JessicaPanagiotidis, Mihalis I.Copple, IanPandey, RenuCorbet, CyrilPanfoli, IsabellaCorke, HaroldPang, Myung-GeolCosme, FernandaPanzarasa, GuidoCosmulescu, SinaPanzella, LuciaCosta, Elisabete V.Paolmino, OlgaCouture, ManonPapada, EfstathiaCouture, RéjeanPapadomichelakis, GeorgeCouzinet-Mossion, AureliePapadopoulos, KyriakosCran, Marlene J.Papież, MonikaCravero, Maria CarlaPapoti, Vassiliki T.Crinelli, RitaPapoutsis, KonstantinosCrisan, GianinaPappa, AglaiaCroft, LarryPappas, ChristosCropotova, JannaPark, Dong SunCross, Carroll E.Park, JunsooCrupi, PasqualePark, Kyoung-ChanCruz, Rui M.S.Park, Shin-HyungCullen, JosephPark, Woong JuneCunha, Luis MiguelParker, Williamcurko, NatkaParsonage, DerekCurti, DanielaPaschalidis, Konstantinos A.Cusanelli, EmilioPaschalis, VassilisCutrignelli, Monica IsabellaPascual-Teresa, SoniaCzerwinska, MonikaPasquale, PignatelliD. Bortner, CarlPasquinelli, GianandreaD??Anneo, AntonellaPastore, AnalisaDa Silva, Anabela BernardesPathak, DhrubaDadan, MagdalenaPatruno, AntoniaDalessandri, DomenicoPaventi, GianlucaDanilenko, MichaelPavic, ValentinaDanjou, Pierre-EdouardPawlak, AleksandraD'Antona, NicolaPazdro, RobertDarvin, MaximPedan, VasilisaDash, SrikantaPeiretti, Pier GiorgioDave, SandeepPelagalli, AlessandraDavies, MichaelPelle, Flavio DellaDavinelli, SergioPellegrino, DanielaDe Bellis, LuigiPeluso, IlariaDe Feo, VincenzoPeluso, Marco E. M.De Filippis, VincenzoPeña-Gómez, NatalyDe Geest, BartPenson, PeterDe Lange, PieterPepe, SalvatoreDe Lucca Camargo, LiviaPereira, Maria De LourdesDe Mendonca, Dina I. M. D.Perestrelo, RosaDe Vendittis, EmmanuelePérez-Gálvez, AntonioDeb, SubrataPeriago, María JesúsDegl'innocenti, DonatellaPerkins, AndrewDehelean, CristinaPerni, MicheleDekker, MarloesPetit, Patrice X.Del Giudice, RitaPetoumenou, Despoina G.Delaney, ChristopherPetrikaite, VilmaDelattre, CedricPetrivalsky, MarekDella Ca', NicolaPetropoulos, Spyridon ADe-Miguel, ManuelPetrou, Athinoula L.Dent, MajaPetruczynik, AnnaDevkota, Hari PrasadPezeshki, AdelDi Carlo, CostantinoPhilips, NeenaDi Donato, MarziaPichardo, SilviaDi Giacomo, SilviaPickering, RaeleneDi Mattia, Carla DanielaPignitter, MarcDi Nisio, AndreaPincemail, JoëlDi Nunzio, MattiaPinniger, GavinDi Pietro, NataliaPinto, Diana CláudiaDi Sotto, AntonellaPinto, TeresaDiaconeasa, ZoritaPinto, UelintonDias, Maria InesPirgozliev, VasilDiehl-Jones, WilliamPirianov, GrishaDifonzo, GrazianaPirintsos, StergiosDikalov, SergeyPirkmajer, SergejDimas, KonstantinosPiro, GabriellaDimauro, IvanPiscopo, AmaliaDinh, Quynh NhuPiscopo, MarinaDobrikova, AneliaPittalà, ValeriaDolowy, MalgorzataPitucha, MonikaDomagała, SlawomirPiva, TerrenceDomenicotti, CinziaPlastina, PierluigiDominguez, HerminiaPluta, RyszardDomínguez-Alvarez, EnriquePodszun, MarenDonato, Rosario FrancescoPoeggeler, Burkhard H.Donnini, SandraPohl, ElenaDonno, DarioPoiana, MarcoD'Orsi, BeatricePolicar, ClotildeDosoky, NOURAPoma, PaolaDoudin, KhalidPons, ToniDrenjancevic, InesPotaczek, Daniel P.Drevet, Joel R.Povey, AndrewDridi, SamiPrado-Cabrero, AlfonsoDrozdz, RyszardPramanik, ArindamDruzynska, BeataPrassl, RuthDu, YonglePrentice, HowardDuarte, Maria PaulaProestos, CharalamposDudas, JozsefPyka, AlinaDuenas, MontserratQiu, BinDunshea, FrankQu, NingDurante, MirianaQuigley, John GDurazzo, AlessandraQuinlan, GregoryDutartre, PatrickQuinlan, Leo R.Dwivedi, ChandraharQuinzii, Catarina MariaDzimira, StanisławRabuazzo, Agata MariaDziurkowska, EwelinaRachek, Lyudmila I.Eamens, Andrew L.Radušienė, JolitaEarnest, ConradRafikov, RuslanEdamakanti, Chandrakanth RRahimi, FaridEdelman, BradleyRahman, AzizurEdge, RuthRai, DilipEeckhoute, JérômeRajauria, GauravEfird, Jimmy T.Rajić, ZrinkaEibes, GemmaRalli, MassimoEkuni, DaisukeRalph, Stephen JohnEl Ghoch, MarwanRamaiahgari, Sreenivasa C.Elgendy, MohamedRamalho-Santos, JoaoEl-Seedi, HeshamRamaraj, PanduranganEndres, KristinaRamírez, CarmenEsatbeyolgu, TubaRamona, PaltineanEscames, GermaineRandall, Stephen K.Escola-Gil, Joan CarlesRanzato, EliaEskin, MichaelRao, ShiwangniEspinosa, FranciscoRapeanu, GabrielaEsteve-Romero, JosepRashidinejad, AliEstevinho, Leticia M.Rawel, HarshadraiEufemi, MargheritaRayner, BenEuler, GerhildReale, AnnaEvans, MarkRebollo-Hernanz, MiguelFabiani, RobertoReddi, Amit R.Faccia, MicheleReed, Miranda NicoleFalque Lopez, ElenaRegazzoni, Luca GiovanniFalsini, BenedettoRegula, JulitaFancello, FrancescoReigosa, Roger J.Fang, MingxiReindl, KatieFang, Yi-PingRelitti, NicolaFaraone Mennella, Maria RosariaRen, JunFarrell, RobertRenna, MassimilianoFasolino, InesRevilla, EugenioFath, MelissaRibeiro, DanielaFato, RomanaRice, Charles D.Fazio, AlessiaRicelli, AlessandraFedoreyev, SergeyRigano, LuigiFedotova, Julia O.Riis, Jenna L.Fedulov, Alexey V.Rijo, PatríciaFeeney, MorganRinnerthaler, MarkFernandes, RúbenRivero-Pérez, M. DoloresFernández Barbero, GerardoRizzello, Carlo GiuseppeFernandez-Ochoa, AlvaroRizzi, FedericaFerretti, GiannaRoca, JordiFeveile Young, JetteRocca, CarmineFia, GiovannaRoche, EnriqueFiacco, ToddRodrigues, António S.Fidalgo, FernandaRodríguez-Roque, María JanethFilep, Janos G.Rodríguez-Solana, RaquelFine, James BurkeRomero, AlejandroFini, Mehdi ARondeau, PhilippeFischer, HenrikeRondonuwu, Ferdy SFischer-Fodor, EvaRosa, DivellaFischer-Schrader, KatrinRoseiro, Luísa B.Fisher, Jonathan S.Rosenfeld, WarrenFletcher, Nicola F.Rosicka-Kaczmarek, JustynaFlorez-Fernandez, NoeliaRoss, IanFontanini, DeboraRos-Santaella, Jose LuisFornai, FrancescoRossi, LorenzoForti, LucaRossi, MiriamFotopoulos, VasileiosRoszak, JoannaFranco, RafaelRouphael, YoussefFranco, RodrigoRoutray, WinnyFrank, SasaRozalski, MarcinFranko, AndrasRozhon, WilfriedFratini, FilippoRubis, BlazejFredotovic, ZeljanaRui, LixinFritz, Kristofer SRuiz, MarioFrolov, AndrejRuiz Téllez, TrinidadFujii, RitsukoRuiz-Aceituno, LauraFujita, MasayukiRupasinghe, ThusithaFulle, StefaniaRusso, AlessandraFurfaro, Anna LisaRusso, DanielaFurue, MasutakaRusso, Gian LuigiFuso, AndreaRusso, MarinaGaascht, FrancoisRusso, Mario VincenzoGai, FrancescoRusso, NinoGaina, Luiza IoanaRybczyńska-Tkaczyk, KamilaGalanis, AlexisRychlik, IvanGalic, AnteRzepecka-Stojko, AnnaGall Troselj, KoraljkaRzewuska, MagdalenaGalvano, FabioRzymowska, JolantaGama, VivianSadowska-Krępa, EwaGambini, JuanSadowski, MartinGan, RenyouSaez, CarmenGanau, MarioSafe, Stephen H.Ganguly, AvishekSagratini, GianniGantner, MagdalenaSaha, AchintoGarbayo, InesSahu, Ravi PGarcia, ConcepciónSaito, SatoshiGarcia, MontserratSakurai, Toshihiro SakuraiGarcia-Alonso, Francisco JavierSalafranca Lázaro, Francisco J.Gareth, NyeSalazar, GloriaGarnacho-Castano, Manuel VicenteSaldanha, CarlotaGarten, RyanSaleh, MahmoudGatti, AntoniettaSalinska, ElzbietaGaweł-Beben, KatarzynaSalmon, AdamGawlik-Dziki, UrszulaSaluk-Bijak, JoannaGazzano, AngeloŠamec, DunjaGebicki, JanSánchez-García, DavidGentile, FabrizioSancho, JaimeGeorgiev, VasilSanjust, EnricoGeorgiou, ChristosSano, TeruoGerber, Leonard E.Sansbury, Brian E.Gerothanassis, Ioannis P.Santacroce, LuigiGescher, AndreasSantos, Lília M. A.Geszke-Moritz, MałgorzataSantucci, AnnalisaGhag, GauravSantulli, GaetanoGherasim, CarmenSanz, AnaGiampieri, FrancescaSaphier, OshraGianotti, AndreaSardu, CelestinoGieseg, Steven P.Satomi, YoshikoGiles, GregorySavion, NaphtaliGimeno-Mallench, LuciaSawicki, RafalGiovannoni, RobertoSawynok, JanaGiovinazzo, GiovannaSchäffner, AntonGIROLAMI, FlaviaSchauss, AlexanderGiromini, CarlottaSchiavone, AchilleGismondi, AngeloSchiavone, MarcoGiuffre, Angelo MariaSchiavone, StefaniaGiuffrida, DanieleSchlaepfer, Isabel RGiuliano, MichelaSchleusener, JohannesGiunta, SalvatoreSchlüter, Klaus-DieterGius, DavidSchmidt, EdGniazdowska, AgnieszkaSchomburg, LutzGoeckenjan, MarenSchöpfer, JuttaGöhre, VeraSchuck, AlyssaGolestaneh, NadySchwartz, Nancy B.Golmohamadi, AmirSchwarz, GünterGomez-Pastor, RocioŚcibisz, IwonaGong, XiaomingScognamiglio, MonicaGonkowski, SlawomirScorziello, AntonellaGonzalez, JavierSeale, Lucia A.Gonzalez-García, JorgeSeca, Ana Maria Loureiro DaGonzalez-Manzano, SusanaSegarra, Ana BelenGorgan, LucianSegatto, MarcoGornas, PawelSehgal, InderGospodarek, JaninaSeiler, Magdalene J.Goswami, Prabhat C.Sekara, AgnieszkaGoto, TakaharuSekiya, MizukiGoua, MarieSelan, LauraGoula, AthanasiaSelvaggini, RobertoGoulas, VlasiosSenbonmatsu, TakaakiGrace-Farfaglia, PatriciaSeo, DaisukeGraczyk-Jarzynka, AgnieszkaSepulveda, Maria Alicia CarrilloGrambow, EberhardSereikaite, JolantaGramza-Michalowska, AnnaSerio, FrancescoGranados-Principal, SergioSerpa, JacintaGrant, GeorgeSerrano, MaríaGreen, ErinSerratosa, María PérezGreenlief, C. MichaelSetzer, William N.Grider, ArthurSgorbini, BarbaraGrieb, PawelShahidi, F.Grimm, BernhardShakeri, MajidGrimm, Marcus O. W.Shankar, EswarGrosso, GiuseppeSharma, AbhisheakGruhlke, Martin C. H.Sharoni, YoavGruzman, A.Shcherbik, Natalia V.Grzela, RenataShen, XingguiGrzelak-Błaszczyk, KatarzynaSheng, RuilongGuerriero, GiuliaShertzer, Howard G.Guescini, MicheleShiao, Young-JiGuido, LuisShih, Ming C.Guillen-Bejarano, RafaelShimanouchi, ToshinoriGumieniczek, AnnaShults, Nataliia V.Gumienna, MalgorzataŠic Žlabur, JanaGunia-Krzyzak, AgnieszkaSicari, VincenzoGuo, Chih-HungSiddique, Abu BakarGuo, FenghaiSiddiqui, Rafat A.Gushina, IrinaSidiropoulou, ErasmiaGutierrez, MargaritaSidoti, AntoninaGuzik, MaciejSieber, FritzHagglund, Per MartenSilaghi-Dumitrescu, RaduHalldorsson, SkarpheoinnSilva, Amelia MHallmann, EwelinaSilva, FilomenaHan, JaehongSimirgiotis, MarioHan, JinsongSimitzis, PanagiotisHanania, MichelSinanoglou, VassiliaHancock, JohnSingh, AnilHandzlik, JadwigaSingh, Ashok K.Hanif, MuhammadSiniscalco, DarioHano, ChristopheSipos, LászlóHansen, Jason M.Skaltza, LianaHara, HideakiSkarková, VeronikaHargreaves, IainSkenderidis, ProdromosHarrison-Bernard, LisaSkoko, John J.Harvey, Patricia J.Skonieczna-Żydecka, KarolinaHasegawa, HiromasaSkouta, RachidHasegawa, MorifumiSkrovankova, SonaHashimoto, NaotoSlominski, AndrzejHassan, MohamedSmeriglio, AntonellaHassan, Sherif T. S.Smieszek, AgnieszkaHealy, Helen G.Sneddon, JenniferHebelstrup, Kim H.Snyder, Nathaniel W.Hegedusova, AlzbetaSobeh, MansourHermesz, EditSobierajska, KatarzynaHernandez, AngelSocaci, SoniaHernandez-Castellano, Lorenzo E.Socha, RobertHernández-Mesa, MaykelSoe, KentHerrero, BaudilioSolano, FranciscoHerrero, EnriqueSoler, CarlesHervás-Marin, DavidSone, HidekoHeydemann, AhlkeSong, Yang-HeonHill, JonathanSoural, MiroslavHinds, Terry D.Sousa, Hélder SHirano, KatsuyaSoveral, GraçaHirst, Jonathan D.Sowa, IreneuszHoffmann, Klaus H.Speciale, AntonioHofmann, TamásSpigno, GiorgiaHollander, John M.Spisni, EnzoHolsinger, DamainSprio, Andrea ElioHoly, RichardSpyridon N., KarrasHolzmann, KlausŠrámek, JanHong, Chwan-YangSrivastava, SaurabhHong, Jiann-RueySroka, ZbigniewHonoré, BentStacchiotti, AlessandraHorowitz, John D.Stadnik, JoannaHoshi, AkihikoStagos, DimitriosHosomi, RyotaStahl, WilhelmHou, Chien-WeiStănciuc, NicoletaHouston, BrendanStanek, MagdalenaHsia, Shih-MinStanley, Jone A.Hsieh, Chang-WeiStanley, RogerHsieh, Ming-JuStarowicz, MałgorzataHsieh, Tusty-JuanStarzak, KarolinaHsieh, Wen-ChingStefania, FilosaHsu, Fei-TingŠtefanić, Petra PeharecHsu, ToddStefano, Antonio DiHuang, Chun-YungStefanon, BrunoHuang, Guan-JhongSteinbrenner, HolgerHuang, Huey-ChunStepanić, VišnjaHuang, Kuo-HowSterczyńska, MonikaHuang, LeiSterken, MarkHuang, Tse-HungStewart, AdeleHuang, Ya-lingStixova, LenkaHuchzermeyer, BernhardStobdan, TseringHuertas, Jesús R.Stojko, JerzyHung, Shao-WenStornaiuolo, MarianoHung, Yu ChiangStout, RandyHwang, Dae YounStowe, DavidHWANG, Gi-WookStrati, Irini F.Hwang, SeongbinStrube, JochenIbeanu, GordonStrzemski, MaciejIbrahim, Mohamed AliStudzińska-Sroka, ElżbietaIbrahim, SalamStuper-Szablewska, KingaIchikawa, HiroshiStushnoff, CecilIida, KaorukoSugahara, TakuyaIkemori, AtsukoSuh, Dae-yeonInoue, AtsukoSuh, Hyung JooInsenser, MaríaSuleria, HafizIohara, DaisukeSuliburska, JoannaIommelli, FrancescaSuntres, ZachariasIqbal, Hafiz M. N.Surai, Peter F.Irakli, MariaSureda, AntoniIrato, PaolaSutliff, RoyIshibashi, KenichiSuzuki, KatsuhikoIshikawa, KenjiSuzuki, Yuichiro J.Ishitsuka, YoichiSvavarsson, Halldor GudfinnurIskandar, Michele M.Svirshchevskaya, ElenaIsola, GaetanoSvoronos, Paris D.N.Ito, ShosukeŚwiątek, PiotrIto, TakamichiSwieca, MichałIvanova, Alla V.Sykłowska-Baranek, KatarzynaIwaoka, MichioSytar, OksanaIzawa, Kazuhiro P.Sytykiewicz, HubertIzawa, TakeshiSzakiel, AnnaJ. Barba, FranciscoSzeiffova Bacova, BarbaraJablonska, KarolinaSzewczyk, KatarzynaJablonska-Trypuc, AgataSzilágyi, LászlóJackson, SarahSzolnoky, GyozoJadeja, RavirajsinhSztanke, MałgorzataJajtner, AdamSzumny, AntoniJakobusic Brala, CvijetaTafuri, SimonaJakubczyk, AnnaTajiri, NaokiJames, Rob S.Takahashi, HirokoJamurtas, AthanasiosTakahashi, MisaJanda, ElzbietaTakano, KatsuraJandeleit-Dahm, KarinTakegahara, NorikoJang, AeraTakemori, HiroshiJara Palacios, María JoseTakizawa, ShinobuJarvenpää, Eila P.Tamasi, GabriellaJavadov, SabzaliTanase, CorneliuJay-Gerin, Jean-PaulTang, BorJeandet, PhilippeTanini, DamianoJekabsone, AisteTarola, Anna MariaJembrek, Maja JazvinšćakTarozzi, AndreaJenssen, HåvardTate, Shin-IchiJeon, SookyoungTatone, CarlaJhun, Bong SookTattini, MassimilianoJiang, Lin-HuaTauler, PedroJiménez-Aspee, FelipeTavazzi, BarbaraJiménez-Castro, Mónica B.Tay, Chor YongJin, JinTaylor, CarolineJohnston, CarolTedesco, DorianaJokic, StelaTedesco, IdoloJones, HuwTeiber, JohnJones, MerielTena, NoeliaJordao, António ManuelTews, DanielJoven, JorgeThackray, AlanaJuhasz, TamasThomas, Dr. RaymondJullian, NathalieThorwald, MaxJurisicova, AndreaTiano, LucaJurkonienė, SigitaTiidus, PeterJurkowska, HalinaTiso, NatasciaJuszczak, LesławTitus, MarkKador, Peter F.Toepel, JörgKafarski, PawelTofani, DanielaKagawa, NatsukoTokars, Valerie L.Kageyama, HakutoTomaszewska, EwaKahlert, StefanTomaz, IvanaKajarabille, NaroaTomczyk, MichałKamdem, DonatienTonetti, LorenzoKamolz, Lars-PeterTonolo, GiancarloKanczler, JanosTosti, ElisabettaKang, Kyo BinTrachana, VarvaraKang, Sang WonTraini, ToninoKang, YunTrentini, AlessandroKao, Chai-LinTrevisan, AndreaKaplan, BarbaraTringali, CorradoKappus, RebeccaTripodi, PasqualeKaras, MonikaTroncoso, A.M.Karcz, DariuszTrost, Paolo-BernardoKasapidou, EleniTrujillo-Rodríguez, María JoséKashfi, KhosrowTsai, Jen-ChiehKasumov, TakharTsai, Kun-LinKatada, KazuhiroTsai, Shiao-WenKatayama, KentaroTsantarliotou, Maria PKatayama, ShigeruTseng, Chih-HuaKato, NorihisaTsopmo, ApollinaireKatsoris, PanagiotisTsotinis, AndrewKauppinen, AnuTsourdi, ElenaKawakami, YukiTufarelli, VincenzoKawakita, HidetakaTundis, RosaKawamura, TakujiTurano, PaolaKawata, KazumiTurner, MarkKayano, Shin-ichiTuttolomondo, AntoninoKe, Liang-YinTyndall, JoelKe, YouqiangUeno, MikinoriKeller, AdrianUgalde, José ManuelKelley, EricUlianich, LucaKello, MartinUline, Mark J.Kennedy, DavidUsai, MariannaKerver, JeanUziel, OritKhajehei, ForoughV Ivanov, AlexanderKhalil, AbdelouahedVaiman, DanielKhatib, SolimanValentová, KateřinaKhosla, AashimaValenzuela, RodrigoKicel, AgnieszkaValero, Marta SofíaKieliszek, MarekValgimigli, LucaKietzmann, ThomasValletta, AlessioKim, BhumsooValls-Bellés, VictoriaKim, Choon YoungVan Horssen, JackKim, Dong JoonVarga, ZoltánKim, DongsukVasicek, OndrejKim, DongukVassalle, CristinaKim, Hyeung-RakVassallo, AntonioKim, Hyun SookVázquez-Tato, M. PilarKim, In-JungVaz-Velho, ManuelaKim, Ji YeonVejux, AnneKim, Jong-SangVenditti, PaolaKim, Ki HyunVerardo, Vito (Italy)Kim, Sung-KunVerardo, Vito (Spain)Kim, Young-MoVerbanac, DonatellaKimura, Ken-ichiVergara, DanieleKishikawa, NaoyaVerhoeven, AdrieKishimoto, YoshimiVerotta, LuisellaKlein, ErikVerpeut, JessicaKleschevnikov, AlexanderVersino, ElisabettaKletsas, DimitrisVeskoukis, Aristidis S.Klettner, AlexaViapiana, AgnieszkaKlimis-Zacas, DorothyVicario, Isabel M.Kmiecik, DominikVicente, JoãoKobayashi, YayoiVieira, Helena L. A.Koch, WojciechVijayakumar, SarathKogut, Michael H.Vilas-Boas, MiguelKohyama, NorikoVinciguerra, ManlioKojima-Yuasa, AkikoVinson, JoeKolodziejczyk, JoannaVirgili, FabioKolota, AleksandraVisioli, FrancescoKomoike, YutaVisscher, ChristianKong Thoo Lin, PaulVitalini, SaraKontogiorgis, ChristosVitetta, LuisKopjar, MirelaVodnar, Dan C.Kosmala, MonikaVon Knethen, AndreasKosters, AstridVoth, DanielKosuge, YasuhiroVuong, Quan V.Kotloski, RobertWada, JunKoulen, PeterWagner, AndreasKouretas, DimitriosWallert, MariaKoutelidakis, Antonios E.Walls, DermotKowalczewski, PrzemysławWan, MurphyKowalska, IwonaWang, Chin-KunKoziorowska-Gilun, MagdalenaWang, Horng-darKozlov, Andrey V.Wang, Hui-minKozłowska (Kozlowska), MariolaWang, JiongweiKrajka-Kuźniak, ViolettaWang, Jong-ShyanKřen, VladimirWang, KankanKrisch, JuditWang, KunKritsiligkou, ParaskeviWang, San-LangKrokosz, AnitaWang, XinyuKrolicka, AleksandraWang, YifeiKról-Kilińska, ŻanetaWang, YuKrupa-Kozak, UrszulaWanrooij, PaulinaKuan, Yu-HsiangWasylishen, AmandaKubatka, PeterWatanabe, MasatoshiKucharska, KarolinaWatanabe, MikikoKues, Wilfried A.Watanabe, NaoharuKukula-Koch, WirginiaWebb, RichardKulp, SamuelWeber, FabianKump, DavidWeber, JohnKumrungsee, ThanutchapornWest, James D.Kunicki, MichalWestwell, AndrewKunz, WolframWhite, RogerKuo, Chia-HungWięcek, MagdalenaKuo, Ping-ChungWiel, ClotildeKupczynski, RobertWigle, JeffreyKurihara, HideyukiWilkinson, JennyKurimoto, KazukiWillems, MarkKurpisz, MaciejWilliams, Noelle S.Kurzawa, MarzannaWińska, KatarzynaKus, PiotrWisnieski, LaurenKuznik, NikodemWłodarczyk, MaciejKwa, Faith A.Wnuk, MaciejKwak, SeonyeongWojdylo, AnetaKweon, Oh-KyeongWojtowicz, AgnieszkaKwon, OranWojtunik-Kulesza, Karolina A.Kwon, Tae-HyukWołosiak, RafałKyathanahalli, ChandrashekaraWong, Boon-SengLa Russa, DanieleWood, ScottLabazi, HichamWrobel, MariaLaboisse, Christian L.Wu, Shu-JuLacham-Kaplan, OrlyWu, Yu-JenLachman, JaromirXin, LingLachowicz, SabinaXu, JingLacina, LukasXuan, Tran DangLaight, DavidYager, DorneLamichhane, PurushottamYamada, SohsukeLandi, MarcoYamakuchi, MunekazuLänger, ReinhardYamamoto, SuguruLanning, NathanYamamoto, YusukeLaquintana, ValentinoYamasaki, HideoLasonder, EdwinYang, Chuen-MaoLatunde-Dada, Gladys OluyemisiYang, JialeLau-Cam, Cesar A.Yang, KunLauro, DavideYang, Wei-hsiungLauschke, Volker M.Yaoita, YasunoriLavandera, Jose LuisYe, LiyunLavdaniti, MariaYe, ZhiweiLaVoy, EmilyYee, Callista StephanieLazzarato, LorettaYeh, Jan-yingLe, Anh VYeh, Shu-LanLebaron, RichardYen, Feng-LinLeclerc, EstelleYokawa, KenLecomte, PhilippeYokoyama, Kazunari K.Lee, Chih-HungYoon, YisangLee, Don–HaengYoshikawa, ToshikazuLee, Hong-JinYui, KunioLee, Huei-JaneYumoto, IsaoLee, Hwa-JinYumura, YasushiLee, Jae WookYurchenko, EkaterinaLee, Jin-KooZabetakis, IoannisLee, Jun-SooZakłos-Szyda, MalgorzataLee, Kyung-WooZalewska, AnnaLee, Meng-shiouZampese, EnricoLee, Sang-HanZamyatnin, AndreyLee, Tae SooZapico, Sara CasadoLee, Tzong-ShyuanZapolski, TomaszLee, Tzu-TaiZarkovic, NevenLee, Yeon SunZarrelli, ArmandoLee, Yun-SilZawada, KatarzynaLeicht, ChristofZhang, QichunLeiva-Brondo, MiguelZhou, BangjunLekka, MalgorzataZhu, JiangjiangLengyel, ImreZielinska, DanutaLeon, FranciscoZingg, Jean-MarcLeonarduzzi, GabriellaZinnai, AngelaLeone, AlessandroZłotek, UrszulaLeone, GemmaZou, PingLescure, AlainZoumpoulakis, PanagiotisLeu, SteveZovko Končić, MarijanaLevart, AlenkaZucca, PaoloLewinska, AnnaZunin, PaolaLezza, Angela Maria SerenaZuo, RanLi, ShuaiZuorro, AntonioLiao, Jyh-feiŻyżelewicz, Dorota

